# Comparing ChatGPT-4 and Human Translation of an Outcome Questionnaire: A Randomized, Double-Blinded Non-inferiority Study

**DOI:** 10.7759/cureus.82525

**Published:** 2025-04-18

**Authors:** Christopher B Sørensen, Anders Gram-Hanssen, Jacob Rosenberg, Jason J Baker

**Affiliations:** 1 Surgery, Herlev Hospital, Herlev, DNK

**Keywords:** artificial intelligence, chatgpt, face validation, questionnaire, translation

## Abstract

Introduction

This study aimed to investigate whether ChatGPT-4 could replace traditional forward-backward translation by humans when translating a questionnaire into another language through a non-inferiority design.

Methods

The questionnaire Abdominal Hernia-Q was translated from English to Danish twice using two different modalities: once with traditional forward-backward translations by human translators and once with ChatGPT-4, resulting in two parallel arms. Each version was randomly assigned to 10 participants who had previously undergone ventral hernia repair. All 20 participants had to evaluate the two translations randomly, both before and after face validation. The participants and the interviewer who conducted the face validation were blinded to the translation modalities. The participants were asked to evaluate readability, comprehension, appropriate use of language, and their overall opinion of the two translated questionnaires before and after face validation on a Likert scale of 1-5 . For each outcome, the non-inferiority limit was set to 1 point on the Likert scale.

Results

Both translations were evaluated by 20 participants before face validation and 18 participants after. Before face validation, relevant and significant differences were found for all outcomes favoring the human-translated version, and most participants accurately guessed the translation modality and preferred the human translation. After face validation, there were no significant differences between the assessments of the two versions of the questionnaire, their guesses were less accurate, and no significant difference in their preference was observed.

Conclusion

ChatGPT-4 may serve as a suitable substitute for traditional forward-backward translation when translating an outcome questionnaire, provided that the ChatGPT-4 translation is subjected to human quality control and is pretested through face validation before clinical use.

## Introduction

When adapting questionnaires into other languages, they should first be translated and validated into the native language of the target population. A common translation method involves forward-backward translation, complemented by expert committee review and subsequent face validation [1-3]. In a scientific context, forward-backward translation has roots in cross-cultural psychology and has later been adopted by medical research [1,2,4-7]. A popular method requires four translators, including an expert in the studied medical field and a researcher to oversee the translation process. It has, however, received some critique and is both time-consuming and costly [3,8,9]. With these issues in mind, we speculated that generative artificial intelligence (AI) tools could perform translations of equal quality to forward-backward translation but in a more resource-efficient manner. Generative AI, especially large language models, has rapidly found its way into various aspects of daily life, but it has also been demonstrated to be an efficient tool in many aspects of research [10]. As of 2024, the most popular large language model chatbot is ChatGPT from OpenAI, which was released in November 2022 [11]. ChatGPT is built upon the large language model known as generative pre-trained transformer (GPT). ChatGPT has previously been demonstrated as an effective research tool, helping researchers write cover letters, introduction sections, and abstracts for scientific papers [12-15].

For this study, we translated the questionnaire Abdominal Hernia-Q from English to Danish [16,17]. Abdominal Hernia-Q is a patient-reported outcome measure for patients undergoing ventral hernia repair, and it includes a preoperative and a postoperative form. The original version was in English with no Danish translation available. Upon planning the translation of the questionnaire, we hypothesized that ChatGPT-4 could translate the questionnaire just as well as humans conducting traditional forward-backward translations.

This study aimed to investigate whether ChatGPT-4 could replace traditional forward-backward translation by humans when translating a medical outcome questionnaire from English to Danish in a non-inferiority design. Readability, comprehension, and appropriate use of language were assessed before and after face validation for each version. The objective was to determine the feasibility ChatGPT-4 as a cost-effective, time-saving alternative to human translation while maintaining equivalent quality through face validation.

## Materials and methods

This comparative, randomized, double-blinded non-inferiority study was reported according to the Consolidated Standards of Reporting Trials extension for non-inferiority and equivalence randomized trials guideline (CONSORT non-inferiority) and the Consolidated Standards of Reporting Trials-AI guideline (CONSORT-AI) [18,19]. The study protocol was uploaded to Open Science Framework before data collection commenced [20].

Study design

For this study, the questionnaire was translated from English to Danish twice, once using ChatGPT-4 and once by human translators [16]. A comparison of the translations was made before and after the face validation process, which involved a series of participant interviews aimed at improving the questionnaires and providing evidence for their face validity. Before the face validation process, participants were asked to complete an assessment form that we created to compare the two questionnaire translations. At this point, a study representative was present to assist the participants in completing the assessment form. After face validation, the participants completed the same assessment form again, but this time without the presence of a study representative. The two versions of the translated questionnaire were presented to the participants in random order.

Translations

**Figure 1:**
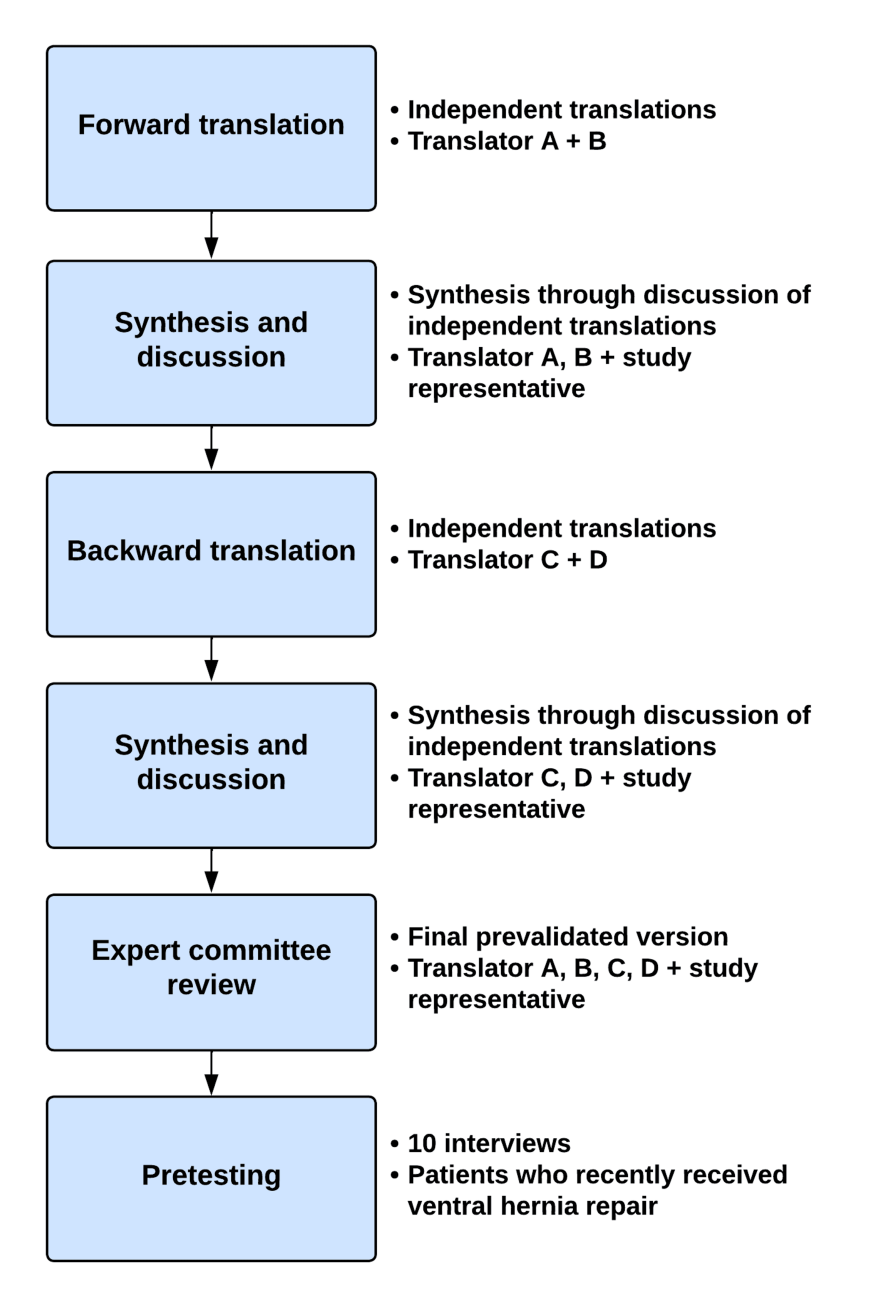
Human translation and validation process Translator A was an expert in hernia surgery, Translator B was naïve in the field of hernia surgery, and Translators C and D were language experts.

The human translation was done using standard-methods of forward-backward translation and expert committee review (Figure 1) [1,2]. Forward translation of the original English version into Danish was done independently by two native Danish speakers, Translators A and B. Translator A was an expert in hernia surgery and Translator B was naïve to the field. Based on the independent translations a collaborative translation was synthesized by Translator A, Translator B, and a study representative. Backward translation from the new Danish version back into English was performed independently by two language experts, Translators C and D, both naïve to the field of hernia surgery. Based on the independent back translations a collaborative back translation was synthesized by Translator C, Translator D, and a study representative. Subsequently, an expert committee review was conducted by the four translators and the study representative overseeing the entire process. The expert committee critically appraised the original English version, the Danish forward translation, and the English backward translation of the questionnaire. Ultimately, a final Danish version was decided on.

**Figure 2:**
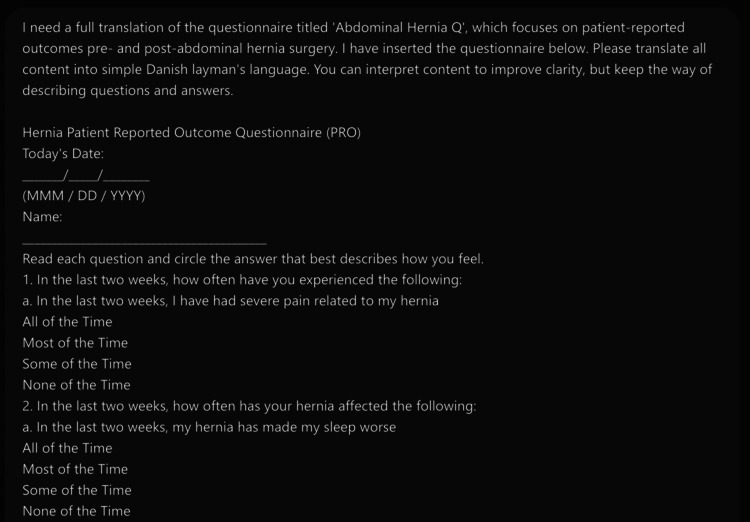
Snapshot of the ChatGPT-4 prompt The prompt has been cropped to include the first two questions of the questionnaire.

**Figure 3:**
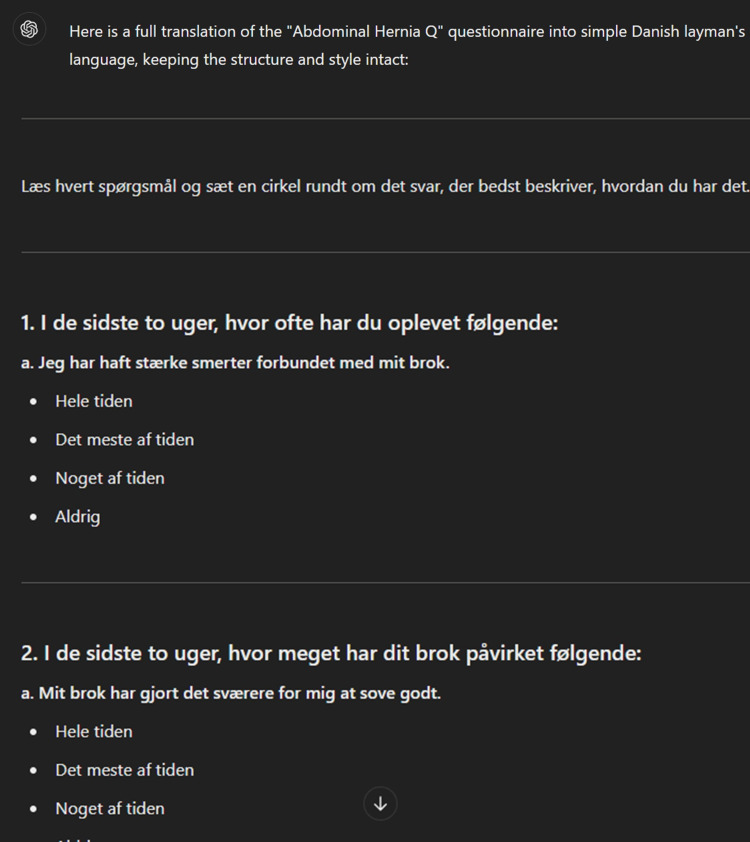
Snapshot of the ChatGPT-4 translation The translation has been cropped to include the first two questions of the questionnaire.

The ChatGPT translation was conducted using ChatGPT-4 via OpenAI’s ChatGPT interface between December 2023 and January 2024 [11]. At the time, the underlying model was GPT-4-turbo, the version available to ChatGPT Plus users after November 2023. Using ChatGPT-4 to create exact translations requires a clear and detailed prompt. The prompt for our ChatGPT-4 translated version went through several pilot tests with varying versions of the wording and the given requirements. During the pilot testing, the setting and context of what the translation should include were described. Several variations of the length and wording were attempted. ChatGPT-4 was also consulted and asked about suggestions for improving the prompts, and the final version of the prompt was based on the context we provided and improved by ChatGPT-4. Earlier versions of the prompt led to variations in output quality, including occasional omissions, hallucinations, and altered meanings. These errors informed the iterative prompt adjustments. The final prompt was selected as the first version that reliably produced a complete translation without interpretive changes or distortions. The version with the best results was: "I need a full translation of the questionnaire titled 'Abdominal Hernia Q,' which focuses on patient-reported outcomes pre- and post-abdominal hernia surgery. I have inserted the questionnaire below. Please translate all content into simple Danish layman's language. You can interpret content to improve clarity, but keep the way of describing questions and answers." This was followed by the questionnaire inserted as plain text in the text input field in ChatGPT-4 (Figures 2, 3).

After the translation processes, a basic quality-control procedure was conducted for both the human and ChatGPT-4 translated versions. For the human translation, this was done through an expert committee review, and for the ChatGPT-4 version, outdated terminology and minor grammatical errors were corrected with the assistance of an expert in the field of hernia surgery. The face validation and human translation process is described in further detail in Sørensen et al. [21].

Participants and sample size

Participants were included in this study if they had received ventral hernia repair and were at least 18 years of age at the time of surgery. Participants were recruited from Herlev Hospital and CPH Privathospital by convenience sampling. Potential participants were contacted by phone successively until we had recruited a total of 20. Of these, 10 participants were involved in the face validation of each version of the translated questionnaire. Participants were contacted by phone and, once oral consent was obtained, an interview was scheduled. During the interview, written consent was obtained. They were excluded if they were cognitively impaired or unable to read or understand Danish.

The required sample size was estimated to be 18 participants. For the sample size calculation, alpha was set to 5%, 1-beta to 90%, SD estimated to 1 point on the 5-point Likert scale, and a one-point difference was considered the minimally important difference. This non-inferiority limit was chosen, as we believed less than one point was not clinically significant. We also wanted to produce a validated questionnaire for future use. For this reason, we decided to use 10 participants for face validation of the human-translated questionnaire, as proposed by relevant guidelines [2]. This meant that 10 participants were also required for face validation of the ChatGPT-4 translation to keep the translation processes equal and maintain blinding of the study representative who conducted the face validation interviews and assessed the results. This resulted in a sample size of 20 participants, which was more than required by the sample size calculation. The sample size was calculated using an online calculator [22].

Outcomes and data collection

The primary objective was to compare the two translated questionnaires after face validation by assessing the readability, comprehension, usage of appropriate language, and the participants’ overall opinions. For this assessment, participants answered four questions using a Likert scale from 1 to 5. Secondary outcomes included which version of the translation the participants preferred and whether they could guess which version was translated by human translators and ChatGPT-4, respectively. The same assessments were done before face validation to determine if the translations were initially equal or if any issues were corrected during face validation.

The comparative assessment included the following questions: "How would you rate the readability of the questionnaire?," "How easy was the questionnaire to understand?," "How appropriate was the language in the questionnaire?," "What is your overall assessment of the questionnaire?," "Which questionnaire do you prefer, and why?," and "Which questionnaire do you think is translated using artificial intelligence, and why?" The first four questions were asked for each questionnaire separately, and were answered using a Likert scale of 1-5, 1 being the lowest rating and 5 being the highest. The last two questions had three possible answers, "Questionnaire A", "Questionnaire B", and "I cannot discern"/"No preference", followed by the option to answer "why" with free text. Face validation was conducted through a series of semi-structured interviews with the participants. The interviews took place in each participant’ private home. During face validation interviews, participants were presented with the following questions: "Is the question difficult to answer?," "Is the question difficult to comprehend?," "Is the question confusing?," and "Is the question causing you discomfort?"

Randomization and blinding

All 20 participants were randomized to participate in the face validation of either the human or the ChatGPT-4 translation. The randomization sequence was generated using an online randomization module [23]. Randomization was done in blocks of four. A non-blinded study representative prepared the presentation order of questionnaires for each interview, and the blinded interviewer was handed two questionnaires named A and B and instructed to present them in a specific order determined by the randomization. Both the interviewer and the participants were blinded from the translation process and were not informed which translation was created by human translation or ChatGPT-4.

Statistics

Data were non-normally distributed and were reported as median and range. Results from the assessments of the questionnaires were not normally distributed. As such they were compared with a one-tailed Mann-Whitney U test. Categorical data were presented as numbers and crude rates and compared with a chi-squared goodness of fit test. Interrater reliability of readability, comprehension, appropriate use of language, and overall opinion of the questionnaire were estimated with Kendall’s coefficient of concordance (Kendall’s W) [24]. Kendall’s W was chosen because this test is suitable test to determine interrater reliability between multiple raters assessing ordinal data. The significance level was p ≤ 0.05 and a significant result meant higher agreement than expected to occur at random. Kendall’s W ranges from 0 to 1, and 0 signifies no agreement and 1 signifies total agreement [24]. Kendall’s W was interpreted analogous to kappa statistics, (0-0.20 = slight agreement, 0.21-0.40 = fair agreement, 0.41-0.60 = moderate agreement, 0.61-0.80 = substantial agreement, and 0.81-1.00 = almost perfect agreement) [25].

Ethical considerations

The study was approved by the Danish Data Protection Agency (p-2023-15228). According to Danish law, this study did not require ethical approval.

## Results

**Figure 4:**
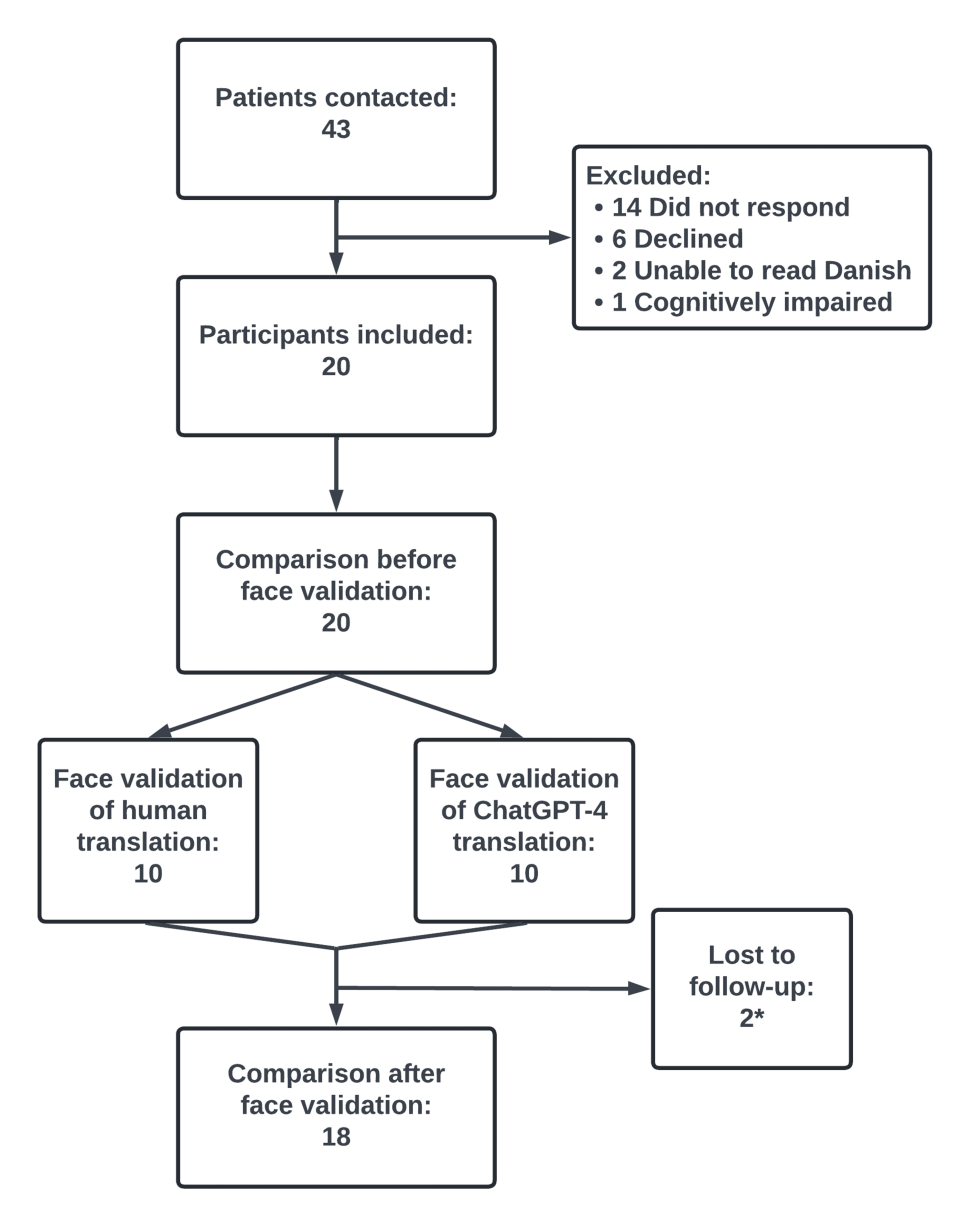
Flowchart depicting study design and participant recruitment *Both participants lost to follow-up face validated the ChatGPT-4 translation

We included 20 participants of the 43 eligible (Figure 4). All completed the first assessment form of the two translations of the questionnaire and the face validation process. Two participants were lost to follow-up and did not conduct the assessments of the final versions of the face-validated questionnaire. One participant did not wish to participate and another failed to reply. Both had participated in face validation of the ChatGPT-4 translation. Participants were recruited in February-March 2024 and follow-up occurred in April-May 2024.

Most participants were male, held at least a bachelor’s degree, and were treated for a primary ventral hernia within the last 12 months (Table 1). All 20 participants assessed both translated versions of the questionnaire before face validation and 18 assessed both translations after face validation.

**Table 1:** Participant characteristics n: Number of participants

Characteristic		Participants (n = 20)
Female sex, n (%)		6 (30)
Male sex, n (%)		14 (70)
Age in years, median (range)		60 (34–71)
Education level		
	Primary education, n (%)	0 (0)
	Secondary education, n (%)	3 (15)
	Tertiary education, n (%)	2 (10)
	Bachelor’s degree, n (%)	6 (30)
	Master’s degree or above, n (%)	9 (45)
Primary hernia, n (%)		13 (65)
Months since operation, median (range)		4 (1–13)

Before face validation, the human-translated version of the questionnaire was rated at least one point higher on the parameters of readability, comprehension, appropriate use of language, and overall opinion of the questionnaire (Table 2). Additionally, most participants accurately distinguished between the human and ChatGPT-4 translations (Table 3) and participants generally preferred the human translation (Table 4). After face validation, there were no significant differences regarding readability, comprehension, appropriate use of language, and overall opinion of the questionnaire (Table 2). Most participants still accurately guessed the translation modality (Table 3) and 50% preferred the human translation (Table 4).

**Table 2:** Assessments of the translated questionnaire Participants’ assessment of the human and ChatGPT-4 translated questionnaires are presented on a Likert scale of 1–5. Statistical analyses were conducted with one-tailed Mann-Whitney U tests. P-values <0.05 indicate statistically significant inferiority in the ChatGPT-4 translation. n: Number of participants

Parameter		ChatGPT-4 median (range)	Human median (range)	p-value
Before face validation		n = 20	n = 20	
	Readability	4 (3–5)	5 (4–5)	0.008
	Understandability	4 (2–5)	5 (4–5)	0.004
	Appropriate language	4 (2–5)	5 (4–5)	0.001
	Overall opinion of questionnaire	4 (2–5)	5 (4–5)	0.002
After face validation		n = 18	n = 18	
	Readability	5 (3–5)	5 (3–5)	0.341
	Understandability	5 (3–5)	5 (4–5)	0.082
	Appropriate language	5 (2–5)	5 (3–5)	0.359
	Overall opinion of questionnaire	4 (2–5)	5 (3–5)	0.100

**Table 3:** Participants’ guesses on translation modality Statistical analyses were conducted with chi-squared goodness of fit test. P-values <0.05 indicate there are statistically significant differences between the participants’ answers and answers picked at random. n: Number of participants

Guess on translation modality	Correct, n (%)	Incorrect, n (%)	Cannot discern, n (%)	p-value
Before face validation, n = 20	18 (90)	2 (10)	0 (0)	<0.001
After face validation, n = 18	12 (67)	4 (22)	2 (11)	0.01

**Table 4:** Participants’ preferred translation Statistical analyses were conducted with chi-squared goodness of fit test. P-values <0.05 indicate there are statistically significant differences between the participants’ answers and answers picked at random. n: Number of participants

Preferred translation	Human, n (%)	ChatGPT-4, n (%)	No preferences, n (%)	p-value
Before face validation, n = 20	18 (90)	2 (10)	0 (0)	<0.001
After face validation, n = 18	9 (50)	2 (11)	7 (39)	0.11

Before face validation, the results from the interrater agreement test found that the participants were in moderate to substantial agreement when assessing the two questionnaires. After face validation, the interrater agreement was fair for comprehension and overall opinion but insignificant for readability and appropriate use of language indicating disagreement for these parameters (Table 5).

**Table 5:** Interrater agreement with Kendall’s W [23] Kendall’s W scores between 0-1, a higher score indicates more agreement. P-values ≤ 0.05 indicate stronger agreement than expected by random occurrence.

Parameter		Kendall’s W	Strength of agreement	p-value
Before face validation				
	Readability	0.417	Moderate	0.004
	Comprehension	0.417	Moderate	0.004
	Appropriate language	0.563	Moderate	<0.001
	Overall opinion of questionnaire	0.650	Substantial	<0.001
After face validation				
	Readability	0.056	Slight	0.317
	Comprehension	0.278	Fair	0.025
	Appropriate language	0.100	Slight	0.180
	Overall opinion of questionnaire	0.333	Fair	0.014

## Discussion

We found that ChatGPT-4 could replace traditional human forward-backward translation of an outcome questionnaire if it was face-validated before clinical use. After face validation, there were no significant differences in the measured parameters, and half of the participants preferred the version translated by humans, whereas before face validation, the human-translated questionnaire outperformed the version translated by ChatGPT-4 in all parameters and was preferred by almost all participants.

Significant differences were observed in all parameters before face validation, suggesting a lower quality of the ChatGPT-4 translation. These findings were supported by another study comparing the translation of legal texts, while other studies found ChatGPT-4 to generate text of equal quality to medical researchers [13,14,26]. However, the lack of significant differences after face validation implies that ChatGPT-4’s shortcomings were corrected through the face validation process. Thus, human involvement is currently a requirement when using ChatGPT-4, and this is also supported by other literature [27].

Many participants were able to accurately guess which translation was written by ChatGPT-4, both before and after face validation. These findings were equivalent to previous studies [13,14]. Additionally, interrater agreement was greater before face validation. The lower agreement after face validation suggests, that while no differences were observed in our primary analyses (Table 2), on an individual level few participants may have perceived the human translation as superior. Based on these participants’ comments, ChatGPT-4 used abbreviations improperly and was slightly less detailed. Thus, adjustments of abbreviations and focus on explanatory text could potentially further improve the ChatGPT-4 translation.

Strengths and limitations

This study had several strengths, including adherence to the CONSORT non-inferiority reporting guideline and calculation of sample size, which ensured the recruitment of an appropriate number of participants [18]. To our knowledge, this is the first randomized, double-blinded non-inferiority trial to assess the feasibility of ChatGPT-4 in medical questionnaire translation, offering a novel, resource-efficient alternative for clinical research. The use of ChatGPT-4 significantly reduces the time and cost associated with forward-backward translation, which traditionally requires multiple translators and expert committee reviews. This has significant implications for resource-limited research settings. Additionally, both the interviewer and participants were blinded to translation modality, reducing the risk of performance and observer bias. Also, risk of inductive bias and effect of order bias were minimized by randomizing the presentation order of the questionnaires for each participant. There are, however, also some limitations. This study compared translation from English to Danish, which may not be representative of other languages and cultures. The generalizability of these findings to languages with more complex grammatical structures or significant cultural idioms remains unclear. Future studies should explore whether similar results can be achieved in non-European languages. Most participants held a bachelor’s degree or above (Table 1), but it does not fully represent the general patient population. The small sample size and overrepresentation of male participants with tertiary education (Table 1) may limit the generalizability of these results to more diverse patient populations. Also, this study was conducted with ChatGPT-4, which, as of May 2024, is no longer the newest version. The performance of ChatGPT-4 is subject to change with updates, posing a challenge for long-term reproducibility. Ensuring future studies document the version used and monitor for changes in output quality will be crucial.

Perspectives

ChatGPT-4 shows promise as a tool for translation, with some limitations such as improper use of abbreviations and a tendency for removal of explanatory text in parentheses. If ChatGPT-4 is used as an alternative to forward-backward translation, we would recommend human quality control prior to pretesting. The shortcomings of the ChatGPT-4 translation were subtle after face validation, and we believe they could be nonexistent without the restrictions within this study. Future studies involving different cultures and languages are warranted to determine the generalizability of these results. Despite the promising findings, it's important to note that ChatGPT’s responses are not always consistent, with previous studies showing fluctuations in its performance [28]. These fluctuations could be a result of software updates. Therefore, ongoing evaluation and refinement of AI translation tools are essential.

## Conclusions

This study demonstrates that ChatGPT-4, with appropriate human oversight, may produce translations of medical outcome questionnaires that are comparable to traditional human translations after a face validation process. While the human-translated questionnaire initially performed better in terms of readability, comprehension, and appropriate use of language, the differences between the two versions disappeared following face validation. These findings suggest that AI-based translation, supplemented by human quality control and face validation, has the potential to serve as a cost-effective and time-saving alternative to traditional translation methods.
